# Empirical evidence for the validity and reliability of resource-use measures based on patient recall: a systematic review

**DOI:** 10.1186/1745-6215-14-S1-P55

**Published:** 2013-11-29

**Authors:** Joanna Thorn, Sian Noble, Theresa Moore, William Hollingworth

**Affiliations:** 1 University of Bristol, Bristol, UK

## Background

Accurate measurement of resource use is required for economic evaluations alongside clinical trials. Despite patient questionnaires commonly being employed, concerns over data quality persist, and there is little certainty about best practices. This review aims to collate the evidence concerning the validity and reliability of resource-use measures based on patient recall and to aid health economists in developing better measures.

## Methods

A search strategy incorporating terms covering healthcare resources, utilisation, patient-reported measures and validation/reliability concepts was applied to the MEDLINE, EMBASE and PsycINFO bibliographic databases. Studies were included if they concerned original research to inform costing studies, and were about patient (or proxy) self-reports of healthcare-related resource use in which a comparator (to assess validity or reliability) was specified. Data on study and population characteristics, type of instrument, recall period, sample size, care setting and administration method were extracted.

## Results

13367 abstracts were identified as potentially relevant. Following abstract and full-text screening, 77 articles published between 1976 and 2011 were deemed relevant. The majority focused on adults (n=58), and nearly half of the articles originated from the US (n=37). Emerging themes suggest that better accuracy is achieved when patients are younger, more highly educated and healthier and are answering questions about inpatient and specialist care.

## Conclusions

There is only a limited amount of validity and reliability information available to inform best practice for resource-use measurement in clinical trials. This ongoing review will identify the gaps, giving a clearer view of where research efforts should be concentrated.

